# Cigarette and alcohol consumption among Colombian migrants and Chileans living in Northern and Central Chile

**DOI:** 10.18332/tid/143088

**Published:** 2021-12-06

**Authors:** Nelson Hun, Alfonso Urzúa, Alejandra Caqueo-Urízar, Antonio López-Espinoza, Diego Aragón

**Affiliations:** 1 Escuela de Nutrición y Dietética, Facultad de Salud, Universidad Santo Tomás, Antofagasta, Chile; 2 Instituto de Investigaciones en Comportamiento Alimentario y Nutrición, Universidad de Guadalajara, Ciudad Guzmán, México; 3 Escuela de Psicología, Universidad Católica del Norte, Antofagasta, Chile; 4 Instituto de Alta Investigación, Universidad de Tarapacá, Arica, Chile; 5 Escuela de Medicina, Universidad de Valparaíso, Valparaíso, Chile

**Keywords:** migration, cigarettes, alcohol, Colombians, Chile

## Abstract

**INTRODUCTION:**

In Chile, the migrant population generally reports lower rates of cigarette and alcohol consumption. However, the migratory process and assimilation of behaviors after extended exposure to the host country could increase the consumption of these substances. The aim of this study was to compare cigarette and alcohol consumption among Colombian migrants and Chileans residing in Chile.

**METHODS:**

In 2019, data were collected from 963 Colombian migrants and 909 Chileans in three cities in Chile. The chi-squared test was used to analyze significant differences in cigarette and alcohol consumption between the groups. Subsequently, the relative risk (RR) and corresponding p-values were obtained.

**RESULTS:**

Colombian migrants had a significantly lower consumption of cigarettes than Chileans (16.6% and 25.1%, respectively). Regarding alcohol consumption, Colombian migrants reported lower consumption than Chileans (43.3% and 48.4 %, respectively ).

**CONCLUSIONS:**

The lower consumption of cigarettes and alcohol by Colombian migrants compared to Chileans is positive for the health of migrants. However, it is advisable to promote health interventions to avoid an increase in the consumption of these substances, especially considering that migrants could assimilate the consumption habits of Chileans.

## INTRODUCTION

Over the last four decades, international migration has grown continuously worldwide. The United Nations Organization projects that 400 million people will be migrants by 2050^1^. Latin America and the Caribbean are not exempt from this reality and have experienced a large increase in intraregional migration^2^. Chile has been the preferred destination for many Latin Americans, especially between 2017 and 2019. The percentage of migrants in Chile increased from 4.4% to 6.6% of the total population^3^. Moreover, the Colombian population residing in Chile accounted for 11.7% of the total immigrant population. Nevertheless, it is important to note that 88.2% of the total Colombian population arrived in Chile between 2010 and 2017^3^, making it a recent migratory phenomenon that requires further study.

The migratory process has been related to negative effects on physical^4^ and mental health^5^, eating behavior^6^, well-being, and the consumption of substances such as cigarettes and alcohol, which are mainly associated with acculturation stress and political, economic, and social uncertainty when facing a new context^7-9^. In addition, some westernized host countries are at a more advanced stage of the smoking epidemic, as is the case of Chile. Therefore, the process of assimilation of the host country’s cultural norms by immigrants may indicate higher cigarette consumption compared to their native country^9^.

With respect to alcohol, stress factors related to inadequate socioeconomic and cultural support, discrimination, poverty, and unemployment have been reported as determinants of alcohol consumption and other drug use^10^. Nevertheless, it has been shown that longer exposure to the host country increases alcohol and tobacco consumption^7,11^. Another fundamental aspect lies in the existence of territories that favor the use of substances such as cigarettes and alcohol, according to the ease of acquiring them and frequency at which they are consumed^7^. In addition, younger generations of migrant descent are more likely to try tobacco products than local youth^12^.

In contrast, identified protective factors against alcohol and tobacco consumption within the migrant population are shorter socialization times in leisure environments, such as parties or clubs, and less frequent relationships with people who use these substances^8^. In addition, the characteristics of the country of origin and not having mixed parents (migrant and local) play an important role^8^. In this context, the aim of this study was to compare tobacco and alcohol consumption among Colombian migrants and Chileans.

## METHODS

### Participants

Data were collected from 1872 residents in Northern and Central Chile in 2019. Of these, 963 (51.4%) were Colombian migrants and 909 (48.6%) were Chileans. The Colombian migrants in our sample consisted of 490 (50.9%) women and 473 (49.1%) men. The Chilean sample consisted of 546 (60.1%) women and 363 (39.9%) men. The age range of the total sample was 18–89 years, with a mean age of 35.4 years (SD=12.4).

The participants were recruited from three regions of Chile: Arica and Parinacota, Antofagasta in the Northern Territory, and the metropolitan region located in the central regions, hosting the capital of Chile, Santiago. It should be noted that the metropolitan region and the Antofagasta region are the two regions with the highest number of visas issued as of 2018^13^. Further details of the sample are provided in the Supplementary file.

### Instruments

The items related to cigarette and alcohol consumption from the structured interview E-TONA^14^ were used. The response format of the items varied from dichotomous to multiple-choice. Some of the questions were: ‘Do you use cigarettes?’, and ‘Do you drink alcoholic beverages?’. The categorization of smoker/non-smoker or drinker/non-drinker is based on the absence or presence of the behavior regardless of its frequency.

### Procedure

This research project was approved by the Scientific Ethics Committee of the Catholic University of the North through resolution 011/2018. The participants were evaluated face-to-face after signing an informed consent. The participants were selected using the snowball technique combined with purposive sampling.

### Statistical analysis

The data were analyzed using IBM SPSS V24 statistical program. Descriptive analyses were performed using frequency distribution. To analyze the existence of significant differences in relation to being migrant or local, the chi-squared test was used. Subsequently, the relative risk (RR) and corresponding p-value were obtained.

## RESULTS

Regarding cigarette consumption, the results indicate that the proportion of participants who consumed cigarettes was lower than those who did not, both for the Chilean and Columbian migrants; 16.6% of migrants and 25.1% of Chileans (p=0.000). When comparing cigarette consumption by gender in the migrants, 19.6% of men reported using tobacco compared to 13.8% of women (p=0.019).

Regarding the consumption of alcoholic beverages, 43.3% of migrants reported consuming alcoholic beverages compared to 48.4% of Chileans (p=0.028).

There were no significant differences in the distribution of the frequency of weekly consumption of alcoholic beverages by group (p=0.066). A total of 82.6% of migrants and 82.3% of Chileans consumed alcohol one to two times per week. A total of 11.3% of migrants and 14.1% of Chileans consumed alcohol three to four times per week. A total of 6.1% of migrants and 3.6% of Chileans consumed alcohol five to seven times per week. In the case of alcohol consumption by gender among migrants, 50.7% of males reported consuming alcohol compared to 35.9% of females (p=0.000).

In addition, when participants were asked if they had been diagnosed with alcoholism or considered themselves to be alcoholics, 2.6% of migrants and 1.0% of Chileans responded affirmatively (p=0.012).

Table 1 shows the relative risk of alcohol and cigarette consumption among Columbian migrants (n=940) and Chileans (n=906) living in northern and central Chile during 2019. It also shows the percentages according to gender and age. The Supplementary file shows the percentages of the Colombian migrant and Chilean participants that declared consuming cigarettes and alcohol according to gender and age range.

**Table 1:** Relative risk of alcohol and tobacco consumption among Columbian migrants and Chileans living in northern and central Chile, 2019

	*Colombian migrants (n=940)*		*Chileans( n = 906)*			*χ2*	*RR (95% CI)*	
	*n*	*Yes %*	*n*		*Yes %*
Do you consume cigarettes?	940*	16.6	906*		25.1	000***	0.595 (0.474 –0.748)	
Do you consume alcoholic beverages?	929*	43.3	903*		48.4	028**	0.813 (0.677 – 0.978)	
	***Age (years)***	***Male***				***Female***		
		***RR***		***95% CI***		***RR***		***95 % CI***
Do you consume cigarettes?	19–25	0.69		0.41–1.17		0.81		0.45–1.46
	26–34	0.72		0.42–1.21		0.52		0.30–0.92
	35–44	0.71		0.42–1.21		0.74		0.43–1.26
	45–64	0.62		0.38–1.03		0.38		0.20–0.72
	≥65	-				-		
	Total	0.67		0.52–0.86		0.61		0.47–0.80
Do you consume alcoholic beverages?	19–25	0.98		0.73–1.33		0.66		0.46–0.95
	26–34	**1.16**		0.89–1.52		0.89		0.68–1.16
	35–44	0.89		0.67–1.18		**1.17**		0.80–1.71
	45–64	0.68		0.52–0.89		0.47		0.30–0.74
	≥65	-				-		
	Total	0.93		0.82–1.07		0.80		0.69–0.93

* Differences in sample size due to non-response.

** p≤0.05

*** p≤0.01.

## DISCUSSION

The aim of this study was to describe cigarette and alcohol consumption among Colombian migrants and Chileans, in Chile. Migrant populations generally report lower cigarette and alcohol consumption rates. However, when these results are disaggregated by means of weekly consumption of alcoholic beverages, the number of migrants who consume alcohol five to seven days per week is approximately double that of Chileans. Although this study did not further explore the diagnostic criteria for alcohol use disorder among the drinking participants included in this study, it is important to note that most of these individuals do not recognize the problem of alcohol consumption. This is consistent for both migrants and Chileans. Nevertheless, although the migrant participant alcohol consumption was double that of the Chilean participants, their recognition of the problem was also double.

On the other hand, the literature reports that there are countries that facilitate the consumption of substances due to their accessibility and affordability^7^. Along these lines, it should be noted that Chile has a higher consumption of alcohol and tobacco than other Latin American countries^15^. Thus, the hypothesis that Chile favors the consumption of these substances should not be discarded, and it would be prudent to study and develop this hypothesis further.

Regarding gender, males of both the migrant and local participants reported higher levels of alcohol and cigarette consumption than their female counterparts. This is consistent with the patterns of consumption observed in both countries individually.

### Limitations

It was not possible to determine the specific number of cigarettes consumed daily. Likewise, no relationships were established with variables inherent to the migration process that could lead to an increase in cigarette and alcohol consumption, such as acculturation stress. It would be advisable to determine the moments in which these behaviors associated with assimilation are adopted and abandoned with respect to the time of residence in the host country.

## CONCLUSIONS

The Colombian migrant participants showed lower consumption of cigarettes and alcohol compared to the Chilean participants. On the one hand, this is consistent with the health effect of the immigrant. On the other hand, it could be related to the ease of acquisition in Chile. Thus, it might be prudent to propose that preventive measures to discourage the consumption of these substances also target the immigrant population. Alternatively, a higher proportion of the Colombian migrant participants showed problematic alcohol consumption, along with a higher proportion recognizing it as a problem, compared to the Chilean participants. Therefore, more studies are needed in this area to explore the reasons for such consumption patterns.

## Data Availability

The data supporting this research are available from the authors on reasonable request.
