# Supplementary material for: Cigarette and alcohol consumption among Colombian migrants and Chileans living in Northern and Central Chile
Source: Tob Induc Dis. 2021 Dec 6;19:94. doi: 10.18332/tid/143088 (PMC8647019; doi:10.18332/tid/143088)
Supplement: Supplementary file 1 [file TID-19-94-s1.pdf]

Supplementary file

Table 1. Sociodemographic characteristics of the sample

		Colombian Migrants		Chileans	
	Total (n=1872)	Male (n=473)	Female (n=490)	Male (n=363)	Female (n=546)
Age (M, SD)	35,4 (12.4)	34,9 (9,9)	35,9 (10,4)	34,4 (13,8)	35,7 (14,3)
Age range	18-89	18-72	18-89	18-79	18-85
Years of residency in Chile (M, SD)	4,3 (2,9)	4,3 (2,8)	4,4 (2,9)		
Years of residency in Chile.	1-19	1-19	1-19		
**Economic Activity**	**%**	**%**	**%**	**%**	**%**
Active	63,2	68,6	59,2	61,1	50,2
Retired/pensioned	2,5	2,0	3,0	3,3	5,0
Unemployed	10,0	11,8	8,6	6,1	4,8
Homeowner	6,9	1,4	11,4	1,1	11,0
Student	15,8	14,0	16,9	26,1	28,8
No information	1,6	2,5	0,9	2,3	0,2
**Monthly Income in CLP***	**%**	**%**	**%**	**%**	**%**
< $100.000	8,8	8,6	9,0	2,8	5,6
$100.000-$300.000	27,6	26,9	28,2	19,7	14,4
$300.000-$600.000	33,9	33,0	34,7	23,4	33,1
$600.000-$1.000.000	16,6	18,7	15,0	27,6	22,8
$1.000.000-$1.500.000	7,4	6,1	8,4	12,5	15,5
>$1.500.00	5,6	6,8	4,6	14,0	8,6
**Educational level**	**%**	**%**	**%**	**%**	**%**
Uneducated	7,1	9,0	5,6	2,5	1,5
Basic/Elementary school	16,5	18,1	15,3	8,7	6,7
Middle/ High school	30,1	27,8	31,9	20,0	30,6
Technic Degree	25,9	27,4	24,9	36,4	26,5
College Degree	20,4	17,7	22,4	32,4	34,8

*Note: M: mean, SE: Standard Deviation, *Chilean Pesos*

Table 2. Percentage of the surveyed population that declares tobacco and alcohol consumption, according to sex and age range

		Migrants				Chileans			
		Male		Female		Male		Female	
	**age**	**n**	**Yes%**	**n**	**Yes%**	**n**	**Yes%**	**n**	**Yes%**
Do you consume tobacco?	19 - 25	16	17,4	12	16,4	35	25,2	37	20,3
	26 – 34	25	18,7	17	11,3	19	26,0	26	21,5
	35 – 44	29	20,9	23	17,0	15	29,4	19	23,2
	45 – 64	17	23,9	10	11,5	30	38,5	39	30,2
	>65	0	0,00	0	0,00	6	42,9	1	4,3
	Total	90	19,6	66	13,8	105	29,2	122	22,6
Do you consume alcoholic beverages?	19 - 25	40	43,5	23	32,9	61	44,2	91	50,0
	26 – 34	79	59,0	63	42,3	36	50,7	58	47,5
	35 – 44	70	51,5	50	38,2	29	58,0	27	32,5
	45 – 64	36	49,3	18	20,7	56	72,7	57	43,8
	>65	0	0,00	0	0,00	8	61,5	10	43,5
	Total	232	50,7	168	35,9	192	54,2	243	44,8

© 2021 Hun N. et al.
